# Ethnography and the digital scenario: a typological scheme of differences and evolutionary trajectories

**DOI:** 10.3389/fsoc.2023.1037359

**Published:** 2023-06-06

**Authors:** Giuseppe Michele Padricelli, Gabriella Punziano

**Affiliations:** Department of Social Sciences, University of Naples Federico II, Naples, Italy

**Keywords:** ethnography in social sciences, digital era, typological schema, ethnographic evolutionary trajectory, pillars of change, role of the researcher

## Abstract

The ethnographic method has been a feature of the social sciences since its inception, and for some disciplines, it is markedly characterized by a strong aptitude for physical field research over extended periods in circumscribed communities. However, with the advent of the digital age, this process has undergone further acceleration, upsetting and partly undermining the solid assumptions on which the ethnographic method had been formed, precisely because in the digital scenario, the assumptions of boundaries of contexts, the agency of scenario, and the need for a long-term field investigation change radically. This conceptual analysis aims at providing an overview of the trajectory of the evolution of ethnographic studies in social sciences by trying to trace the main pillars of change and the future direction of the method.

## 1. Introduction. Ethnography to the evidence of a digital turn

The rise of the digital issue has been guaranteed in the last 20 years as a not insignificant frame for social science because of its power of identity building, information, and knowledge sharing in the architectures of relations and networks made by users via computer-mediated communication (CMC).

Beyond this, the standardization of Internet, blogs, social media, and other web-sphere use, is similar considering the offline ways to access information and what concerns the observation of social phenomena (Airoldi, 2017). In-person or web-mediated situations can in fact equally be considered “information systems” (Meyrowitz, 1985) that ≪affect the kind of knowledge we can produce as researchers/observers≫ (Airoldi, 2017, p. 9).

Starting from the idea of the innovation intended as a cultural object composed both as an instrumental repertoire and as well as a set of practices applied by the tools contained in this repertoire, “the various identities, practices, values, rituals, hierarchies, and other sources and structures of meaning that are influenced, created by, or expressed through technology consumption” (Kozinets, 2019, p. 621) have changed over time. Consequently, social research methods are also continuously challenged (and rightly so) with discussions and reflections about the changes related to current innovations tied to the social context in which we live.

In light of this, following Lupton (2018), we cannot agree with the idea that old-fashioned methods, such as ethnography, must be ≪left aside to give more space to those new methods that differently are featured by the current evolution of technologies≫ (p. 41). Sociologists today should instead consider how different approaches can be adopted, what opportunities they open in terms of knowledge production, and what kind of data they produce in order to ≪open a fruitful debate concerning the own nature of our discipline and its future in the digital era≫ (*ivi*).

Quantitative and qualitative methods remain, in fact, so relevant in the current state of social research to investigate identities, daily life, institutions, the social gap, and so on, not to mention investigating whether the opposition of this classic dichotomy has also been overturned because of the implications that technologies carry out for research practices. It is enough to think of the online tools ethnographers today can use as digital recorders during the interviews or the use of software to analyze the new kind of digital data. *User-generated content* (UGC), users' activities on the web-sphere, e-commerce orders, and the whole big-data frame are some examples of new data that is possible to gather from sources not available before the digital turning point.

The emerging epistemological tasks and ethnography, one of the oldest and most consolidated methods in the social research sphere, is no exception in challenging social researchers to rethink their work. Innovations and new technologies bring the standard anxiety from which social researchers have not been excluded across the *digital turn* (Marres, 2017). Many scholars in recent years have been interested in high levels of the administration of social science research methods via the Internet. They were driven toward the idea of ≪new technologies presented both as an opportunity to be grasped and a threat to be countered≫ (Hine, 2000, p. 4). After stating that, it becomes helpful today to reassess the current opportunities and threats for researchers who move inside and outside online environments in light of the epistemological and methodological changes over the past few years. Starting from these assumptions, it becomes relevant to assess the role of technologies in research methods in order to understand the following:

- if that anxiety emotion by which nothing could be taken for granted has been eventually overturned thanks to the running-in of online and offline research methods;
- how the complementarity vocation of online and offline methods helped compensate for what can be lost during ethnographic sessions on physical and non-physical spaces.

Following the plentiful literature, ethnography is intended as a method based on direct observation (Spradley, 1980; Gobo and Marciniak, 2016) that usually involves “the researcher participating, […], in people's daily lives for an extended period, watching what happens, listening to what is said, and/or asking questions through informal and formal interviews, collecting documents and artifacts” (Hammersley and Atkinson, 2007, p. 3).

The main disciplines that were historically suited to direct observation, anthropology, and sociology, are mainly related to the specific focus elected as the fundamental connotation of the ethnographic method: community, field, and the relationship between researcher and researched (Hammersley, 1990). Brunt (2007) retraced how the concept of community has evolved in recent years, first intended as the association between places and people sharing specific interests, feelings, behaviors, and objects (Warner and Lunt, 1941). It is based on the Fletcher formula (Fletcher, 1971) that connects family, work, and places, duly expressed as a ≪group of households situated in the same locality and linked to each other by functional interdependencies which are closer than interdependencies of the same kind with other groups of people within the widest social fields to which a community belongs≫ (Elias, 1974, p. XIX).

Relating to the epistemological assumptions it becomes relevant to the reflection about the role of researchers in the way that ≪For a long time, no one thought much about how fieldwork was written up into descriptions of other cultures≫ (O'Reilly, 2007), neglecting the research outcomes from the researcher's written fieldwork and how the social construction of the reality depends on ethnographers' choices and their narrations. Several epistemological debates have been developed concerning the various ways to approach the ethnographic vocation (such as realism, interpretivism, relativism, etc.). The *reflexive turn* of 1980s and 1990s, was characterized, among others, by Atkinson (1992) and Clifford and Marcus (1986) and drove toward a brand new pragmatic vocation proposed by Hammersley (1998) by which ≪rather than attempting to reproduce reality in our ethnographic accounts we admit the best we can do is to make attempts to represent it≫ (O'Reilly, 2007, p. 184).

The emergence of critical ethnography (Thomas, 1993) explicated the role of social science in doing ethnography, extending the conventional application of the method through the ethnographer's choices. Focused particularly on political issues, the critical vision had already highlighted how the representational consequences of research cannot be considered neutral. On this assumption, postmodernist ethnographers (Burawoy et al., 2000) drove the debate toward the possibility of “evoking” reality rather than “representing” it. It led the contemporary discussion to the idea of an ethnography conducted by human beings who ≪make choices about what to research, interpret what they see and hear, decide what to write and how, and that they do all this in the context of their own personal biographies and often ensconced in scientific and disciplinary environments≫ (Spencer, 2001). This gave up the presumption of being objective and following the attempt to create ethnographies that reflect the complex nature of reality (Hammersley and Atkinson, 2007) and sustaining how the latter exists externally to our possibilities of experiencing it.

In light of this premise, O'Reilly (2007) redefined the assumptions about the ethnographic epistemology debate clarifying that the application of the method consists of ≪iterative-inductive research (that evolves in design through the study), drawing on a set of methods. It involves direct and sustained contact with human agents within the context of their daily lives (and cultures), watching what happens, listening to what is said, asking questions, and producing a richly written account that respects the irreducibility of human experience and that acknowledges the role of theory as well as the researcher's own role≫ (p. 52).

However, when the ethnographic method is tested in the digital environment, most of these assumptions undergo evolutions and revolutions. The initial function of the method framed on a specific object changes its configuration in terms of the place to carry out the ethnographic work due to the subsequent evolution of the instrumentation adapted to the changed scenario.

New methods offer a wide range of new investigation possibilities, but they also have their own limitations and boundaries that social researchers need to experience to make the most of online as well as traditional research methods. Many scholars stated the strict connection between research objects and research questions to study certain phenomena through the most appropriate methods (Phillimore and Goodson, 2004). Following Addeo et al. (2021), a compelling statement that has been the core of social research practice since its beginning is still valid nowadays in the era featured by the so-called *digital turn*.

It is enough, for example, to think about the challenges in exploring new kinds of spaces no more related to physical fields of observation and those social phenomena first transposed, and then, in some cases, totally migrated online. In this way, the compresence of the researcher and research in the same kind of space is not required. At the same time, the compresence does not affect the ethnographical application. Following Schrooten (2016), in fact, ≪the everyday lives of many individuals often transcend the geographical locations in which classical fieldwork took place, challenging ethnographers to include these social spaces in the demarcation of their fieldwork sites≫ (p. 66). In this way, the vision of Hine (2006) concerns the anxiety about how far existing research methods are appropriate for technologically mediated interactions due to the willingness to incorporate the Internet as part of “the field”. It brings us to consider digital technologies and wonder how ≪the study of these sites has substantially increased the range of possible relationships involving fieldnotes≫ (Jackson, 2016, p. 51).

Following Paccagnella (1997) and Di Fraia (2007) digital tools can be intended as flexible objects that enable knowledge production and knowledge sharing. This attitude is still valid in the frame of social research and ethnographic application also by the ≪implementation of practices not provided by developers≫ (p. 23). Thus, we can intend the Web not as a ≪sum of sites but rather as a series of digital resources related to events, concepts or relevant topics≫ (Schneider and Foot, 2011, p. 2) in which ≪the use practices can follow some evolutional paths≫ (Vittadini, 2018, p. 15).

Research actions and their relevant practices follow different paths in data gathering procedures, taking care of technologies and digital tools which can enable researchers to reach their purposes. As already assumed by Padricelli et al. (2021), multiple turning points mark differences and exceptions in every kind of application practiced. Among them, it is enough to think about the relevance of the field notes to collect regardless of the kind of data used (digital, digitalized, etc.) and the opportunity to build primary data or collect secondary data for primary use for social media analysis. On these bases, technologies and digital maps play different roles in doing ethnography. On the one hand, they play an exogenous role in traditional ethnographic applications in the way that, for example, the use of audiovisual recording tools during the observing or interrogating sessions can be useful to transform and re-adapt the method in order to enrich, complement or rearrange fieldnotes by different immersive research experiences.

On the other hand, technology plays an endogenous role in a netnographic way. In this vision, technologies take part of a whole digital context that becomes an additional and integrated social participatory environment where the researchers take into account as well the role they play in relation to technologies and web affordances: researchers that use some data collection tools to access digital fields that are not limited to study the online cultures, but rather that can aim at detecting cultural changes and social conditions through technologies. In this way, following Tummons (2020), the boundaries of the non-traditional applications in ethnography ≪are discursively constructed rather than bounded within geographic spaces≫ (Liu, 2022, p. 3) in the way that ≪digital platforms are both tools and fields to study social relationships that differ from those occurring at traditional sites such as schools, firms, and classrooms. While the research subject may be the same, how researchers “gaze upon” them differently, depending on how technology mediates or highlights a particular dimension of social interactions≫ (*ivi*).

Based on these factors, the time has come to retrace the evolutionary trajectory of the ethnographic practice to provide (young) researchers with a systematization of recent ethnography development so as to know better, threats, limits, and opportunities in choosing the best research path and the method layout to achieve their goals.

Ascertained by this background, the following article aims to investigate the current developments in ethnographic practice to understand its evolutionary trajectory starting from the following research questions:

- How did the digital context change the canonical application of social science?
- How do researchers move inside and outside the online field availing (or not) of research innovations and related digital technologies?

Primarily, the topics presented will focus on the evolution of ethnographic practice in the digital age. Subsequently, the main dimensions of the intervening changes will be reviewed, and a proposed systematization of a typological scheme will be attempted. Finally, we will discuss the emerging pillars on which to focus, to adequately answer the questions that drive this study and its ultimate aim of understanding what happens to the epistemological and ontological essence of the ethnographic method.

## 2. The role of technologies and the trajectories of ethnography

The Internet has developed drastically and has influenced our daily routines, way of life, how we express ourselves, our culture and shared beliefs, knowledge, and ideas. Consequently, the Internet revolution has profoundly impacted ethnography (Garcia et al., 2009) and more generally all methods of investigating cultural and social phenomena. The Internet has made it possible for any researcher to simultaneously access online information, actions, interactions, communities, and cultures located in different places, and then to designate several variations in the application of the method characterized by new advantages and limits concerning the relationship between the field and the researcher, the levels of intrusion, the research actions, and the techniques used (Padricelli et al., 2021).

The turning point for ethnography in light of Internet studies coincides with the new centrality assumed by the concept of cyberspace, beginning in 1990 (Woolgar, 1996) and intended as a place to store large amounts of helpful information to discover how much social culture is online. Cyberspace can also be understood ≪as computer-mediated contexts intrinsically related to supposed-to-be “real” places. From this point of view, the ethnography of online groups is not just the ethnography of the groups online (or the online ethnography of groups). However, it is both the ethnography of online and related off-line situations, the ethnography of humans and non-human actors in these related fields≫ (Teli et al., 2007). This attempt at linking what is on the Internet and what is moving in the world, outside the Internet, concerns the Web not only as a cultural context, but also as a cultural artifact, a flexible, dynamic, and pervasive object. On this requirement, Hine's vision (2000) concerns how research methods need to be continually adapted to the social context, social phenomena, and their characteristics. In this way, the ethnographic method adapted its traditional vocation to the well-known version called “netnography”. Following Hobbs (2006), it consists of a repertoire of practices needed to understand a particular culture. Traditional research methods move to the web environment where real communities become web-communities to preserve, or create, substantive networks and relationships in cyberspace. Based on these assumptions, netnography, upon its inception, was defined as the online transposition of classic techniques: in the same way, as the survey becomes a *web-survey*, the interview becomes a *web-interview*, and so on. Observation is elected as the main research method, complemented by a series of research actions that produce ancillary sources of information such as passive listening, querying, and reading, by which the researcher is not forced to be involved in web-based activities. However, it can instead select a specific level of participation. The assumption related to the observation is also valid for netnography, even if it differs for any feedback effects related to the different observing scenarios addressed online. Netnography can deal with a non-intrusive level: a setup that indeed entails further reflections about the ethical ways of doing research.

On the other hand, for in-person ethnography, the observation concerns an interactive relation between the ethnographer and observed subjects in any case. In this way, we must always speak of intrusion level as well as in case of covert observing sessions by which the observed subjects are unaware of the researcher's identity and purposes (Amaturo, 2012). Beyond all of this, as demonstrated, netnography is not only characterized by a technical emphasis related to comprehending new socialities through online fieldwork. Recently, new definitions have helped to better understand the elaborate epistemological concerns of netnography, intended by Kozinets (2020) as a “set of general instructions relating to a specific way to conduct qualitative social media research using a combination of different research practices” (p. 7).

These practices bring to attention the first dimension of differences it must consider, that is, those that distinguish traditional ethnography and its applications from the netnographic practice based on data-gathering procedures. This dimension of difference immediately recalls the interconnected opposition between immersive and investigative practices in implementing ethnographic research. In detail, *immersion* “references the netnographer's self-reflective and introspective collection of research observations and experiences” (Kozinets, 2022, p. 107). This means that User generated contents (UCGs) available on the web represent real traces to be used as a basis for observation and reflection as well as the continuing relevance of field notes, diaries, or memos, produced by the researchers during the observation of a participatory and co-construct reality. The freely available and directly accessible information from UGCs becomes data that allows for economic savings and faster elaborations. More specifically, the processes of entry, storage, and management of such data are simplified (Acampa et al., 2022). The rise of *user-generated data* is one of the most useful examples of the progressive and rapid evolution of the Internet background. Research methods have not been unaware of its evolution, in fact, in the last few years, social researchers have begun to wonder if and how traditional methods, and their applications in overcoming simple digital transposition, were exposed to moderate gains in terms of costs and efficiency, as well to threats in terms of the quality of data, loss of representativeness, absence of feedback, and validation of the results. The empirical opportunity related to UGCs opens up a second practical declination in netnography defined as *investigation* which refers to the “disciplined collection of already existing data—also called online traces—which, in most netnography to date, has come from the archives of social media platforms, blogs, and forums” (Kozinets, 2022, p. 107).

This vision matches with a transversal dimension of differences that deals with the definition of context and its progressive change which sees the Web go from a communication and information medium to an environment for mediated interactions among individuals; between researchers and individuals, in-depth interviews or other “interpersonal data collection methods such as digital diaries or mobile ethnography” (*ivi*); between researchers and non-human social actors. This environment comprises its ontological artifacts that Patel (2013, p. 411) defined as a “*read-write web”*, “*people-centric web”*, and “*participative web”*. The Web becomes a scenario, currently recalled with the digital locution *scenario*. As with every scenario, it is governed by rules, contains within itself the means that make action conceivable, and defines the spaces within which action is possible, pursuable, and takes on meaning. Following the idea of the Internet intended as an innovation composed of two elements that mutually evolve (an instrumental repertoire as well as a set of practices needed to use the devices), the scenario concept helps to renew the interpretation of the Internet as a space. It needs to consider the double composition of the Internet as a combination made by technical infrastructures and the set of contents it carries, better known as the Web (Gallino, 2003; Grimaldi, 2005). The digital scenario described in this way draws paths that overcome the reduction of the Internet to merely a medium for communicating and spreading information. The mediation process allows opportunities for users to *be* media. It has passed the mediatization process by which it is possible for users, as well as for social researchers, to become media (Boccia Artieri, 2012) through the interiorization of proper codes, aesthetics, and expressive forms which can generate a sense which was not identifiable before the advent of *the digital turn*. This means that it is only possible to study what happens inside the scenario, especially if it is digital, if the researcher takes on the perspective of those inside the scenario. Therefore, it is only possible to produce knowledge on what happens on the Internet if it becomes both an object of study and a methodological tool to investigate it. The ethnographic method better known as *digital ethnography* (Murthy, 2008) follows these assumptions as an approach that makes it possible to recreate a new Internet story from the inside of the device and its own agency ≪linking the researcher directly to the spaces within the studied subjects move and analyzing every relation cluster not concerning the subjects in a place as the virtual world≫ (Consolazio, 2017, p. 81). These points have challenged the solid assumptions on which the classical ethnographic method has been supported. The digital environment with its prerogatives places at the center another dimension of difference that is expressed in overturning the concept of community, making borders more and more fluid, and creating temporary associations and cooperations among strangers with mutual agendas which disappear after a few hours of intense shared experience (Arvidsson and Caliandro, 2016). This assumption of the circumscribability of social actors challenges the need for the researcher to have prolonged exposure to a digitally transposed field: a field configured as something in which everything persists from the moment it is released onward. The mediation function of the technologies is not the only one related to ethnographic approaches. Baulieu (2012) has already identified multiple functions that technologies can assume for research exploration purposes. They transcend the mediatic concerns and twist with the interaction assumptions, taking care of the researcher's position and her/his intrusive or unobtrusive opportunities along the ≪participant/observer *continuum* technology make possible≫ (p. 149). The unobtrusive one in fact enables one to ≪gather the material at the ethnographic level (at the level of specific interactions) without the intrusiveness of the tape recorder or the disturbing physical presence of the observer≫ (Baulieu, 2012, p. 146), while the former, taking care of the socio-technical spaces already defined by Wakeford (1996)—information, communication, and interaction spaces—relate to the human and non-human subject (users, as well as search engines; blogs, website, etc.) (Baulieu, 2012, p. 149).

Following the paradigmatic assumption related to the current mixed-method vocation, today's ethnographers can benefit from the opportunities emerging from physical and digital scenarios related to traditional ethnography and netnographic orientations. The immersive movement inside and outside the digital scenario is a ≪current essential need for researchers to comprehend social phenomena≫ (Punziano, 2022, p. 290) that allows integrating different insights coming from the observation in a (non) digital environment. It is featured by different ways to access and take positions inside the field(s) and by the kind of data used. A recent example of research by Addeo et al. (2021) makes it clear how netnography and traditional ethnography applications can no longer be considered the extremes of a *continuum*. Instead, they must be rethought as methodological practices that enable gradual and intermediate choices based on the research objectives and expected results. They first aim to discover the exclusive or coexisting methods in hybrid ethnographic practices. In this way, current social conditions have been an appropriate opportunity for researchers to test the research question because of the restrictions and limitations related to the COVID-19 pandemic which inhibited mobility and the usual ways of accessing physical places where social phenomena happen. Their study aims to understand the motivation behind visiting *places of suffering* for *dark tourism*.

According to Quarantelli (2000), and the classification of the pandemic as a disaster, researchers have investigated how *dark tourists* can fulfill their tourism desires when they are unable to physically visit *places of suffering*. In February 2020, COVID-19 reached Europe, in northern Italy. In <24 h two small cities close to *Lodi* (*Codogno* and *Vo' Euganeo*) became *off-limits* areas patrolled by police. Over the same 2 days, on social media, many *non-local people* in different Italian regions joined Facebook groups originally created by *Condogno* and *Vo' Euganeo* citizens to share and promote their local activities. Their research consisted of a 3-month non-intrusive observation of non-local interactions. At the end of the observation period, the data collected consisted of 47 posts made by 25 of the 111 non-local users identified. These contents mainly promoted support and charity providing protective equipment and preventative supplies such as masks or sanitizing lotions. The other 86 profiles had yet to interact with local people, positioning themselves as hiding the real reasons for their presence in those Facebook groups. By using an exclusive single-netnographical practice, the researchers could not achieve the expected results and could not comprehend the real motivations of these users to transpose their *dark tourism* experience online.

The integration of the results obtained through the phases of immersion and investigation made inside and outside the (non) digital fields has been quite showed reasonable demonstrated as well as by a recent study made by Padricelli (2023) related to the longitudinal framing reconstruction of the Italian social movement *No Tav*. In this case, a proper netnographic application oriented to the study of digital self-constructions was made by the collective actors on social media during the last 10 years, and the researcher got more interesting results due to an ethnographic exploration by the interviews of activists. The first results concerned how *No Tav* used social media to spread and inform about diagnostic and prognostic reasons for their claims. These have been later integrated into collecting field notes concerning the direct experiences of activists in direct actions led on *the field of civic action* as well as on *strategic action fields* (Postill, 2017). This shows how the mixed method vocation inside and outside the digital field could return expanded results not retraceable by a single online application.

A typological scheme is proposed to better systematize the reflection that emerged from previous examples and, at the same time, have a proper useful instrument to systematize the next correlated ethnographical explorations.

**Figure 1:**
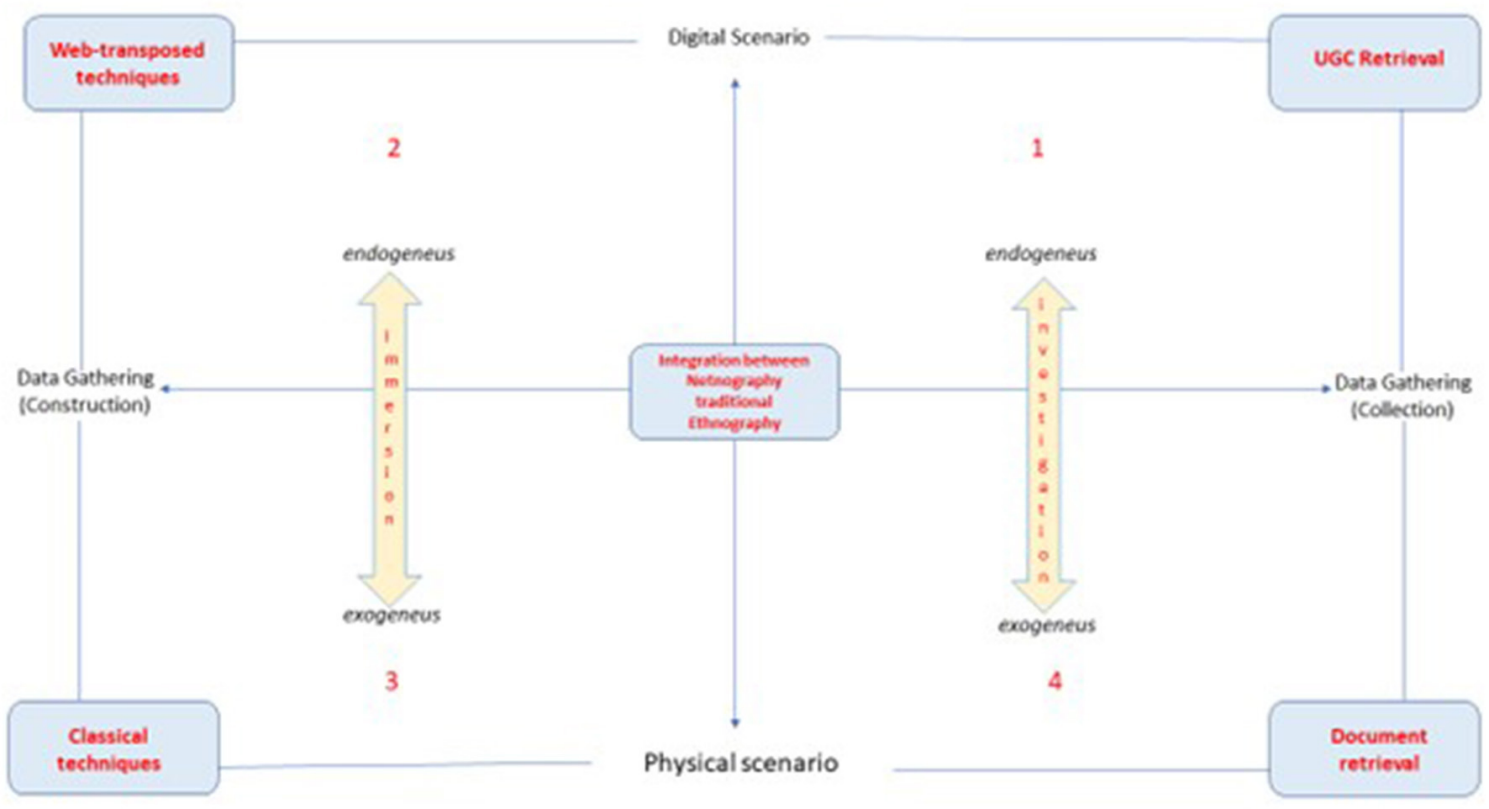
Typological schema of ethnographic trajectory in the digital scenario.

The latter is built, taking care of the main epistemological dimensions previously approached. As shown in Figure 1, the first axis, the horizontal one, underlines the theoretical *continuum* that places the observing scenarios on opposite sides next to the online-offline fields that lie in our first dimension of difference, opposing *physical scenario* and *digital scenario*. The second axis, the vertical one, opposes the different ways of gathering data: on the one hand, data obtained through the construction practices in direct interactions with the research subject, and on the other hand, data obtained by collection procedures taking into account the endogenous or exogenous roles of technologies.

## 3. Imagining a typological scheme: the researcher's movement toward the integration of practices

Looking at the graphic, the second quadrant crosses digital scenarios and data-gathering construction practices. When the researcher takes position here, they can gather information via web-transposed techniques. The latter concerns all those applications of the method featured by observing and interactive action held online because of the presence of researched objects in non-physical backgrounds, such as social media. In this way, as already described in Addeo et al.'s (2021) study, the non-compresence of the researcher and focused subjects is not a condition that affects the success of the research. It can instead raise obstacles in terms of gained results, as the underlying behavior of non-local users in the experimental Facebook groups.

**Figure 2:**
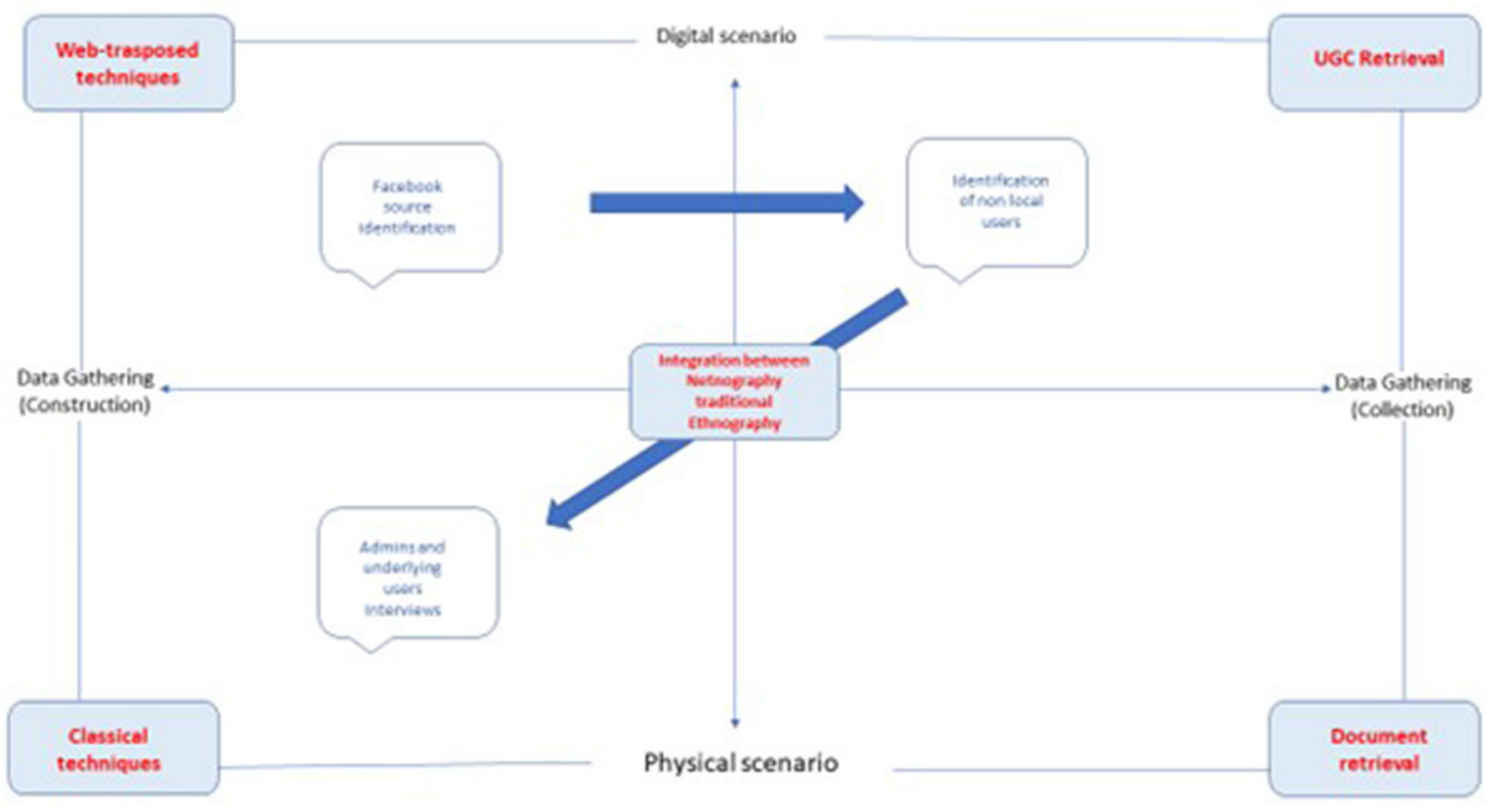
Typological schema applied to *dark tourism* study.

Shifting down to the third quadrant, which crosses data gathering construction practices and physical scenarios, the condition for ethnographers' position in the fields concerns their compresence in the same place as what they observe, despite what concerns the immersive practice featured by netnographic application. In this way, classical techniques related to the application of traditional ethnography (interviews, direct observation, focus groups, etc.) are used for an additional research phase of the research design. It pushes the researcher to join the proper integration in the immersive hybrid scheme. It aims at going into detail and adds more helpful information not traceable by the only single application of netnography. This could be a follow-up of the mentioned study on *dark tourism* (Figure 2). Due to the sensitive topic approached by Addeo et al., formal direct interviews of underlying users, Facebook groups admins, or any citizens located in *Codogno* or *Vo' Euganeo* can turn back interesting, deepened results related to the push factors of digital *dark tourism* experiences they made. In light of this, as shown in Figure 2 by the double direction of the arrow across the second and third quadrants, it is stated that the integration of both techniques can be used starting from the digital scenario and the physical one.

Moving on the right side of the plan, the fourth quadrant concerns the data collection procedures researchers can follow by reading documents and any traces left by the observed subjects. When the researcher takes position in this quadrant featured by the traditional application of the method in the physical scenario, we must consider all the physical documents they can collect or receive directly from the intercepted subjects. Due to the study of documents retrieved, the researcher here positioned can find help to comprehend how social actors are placed in the social context to which they belong. This often occurs through analytical procedures such as content analysis. Moving up in the first quadrant which crosses data gathering via collection procedures in a digital scenario, the researcher here positioned can follow the same investigative assumptions as in the third quadrant, but not being in compresence in the same place as the observed subjects and taking care of the UGCs that can help them to plan framing analysis. They can arrange the study of observed subjects' narratives or discourses on the web-sphere as blogs or social media.

**Figure 3:**
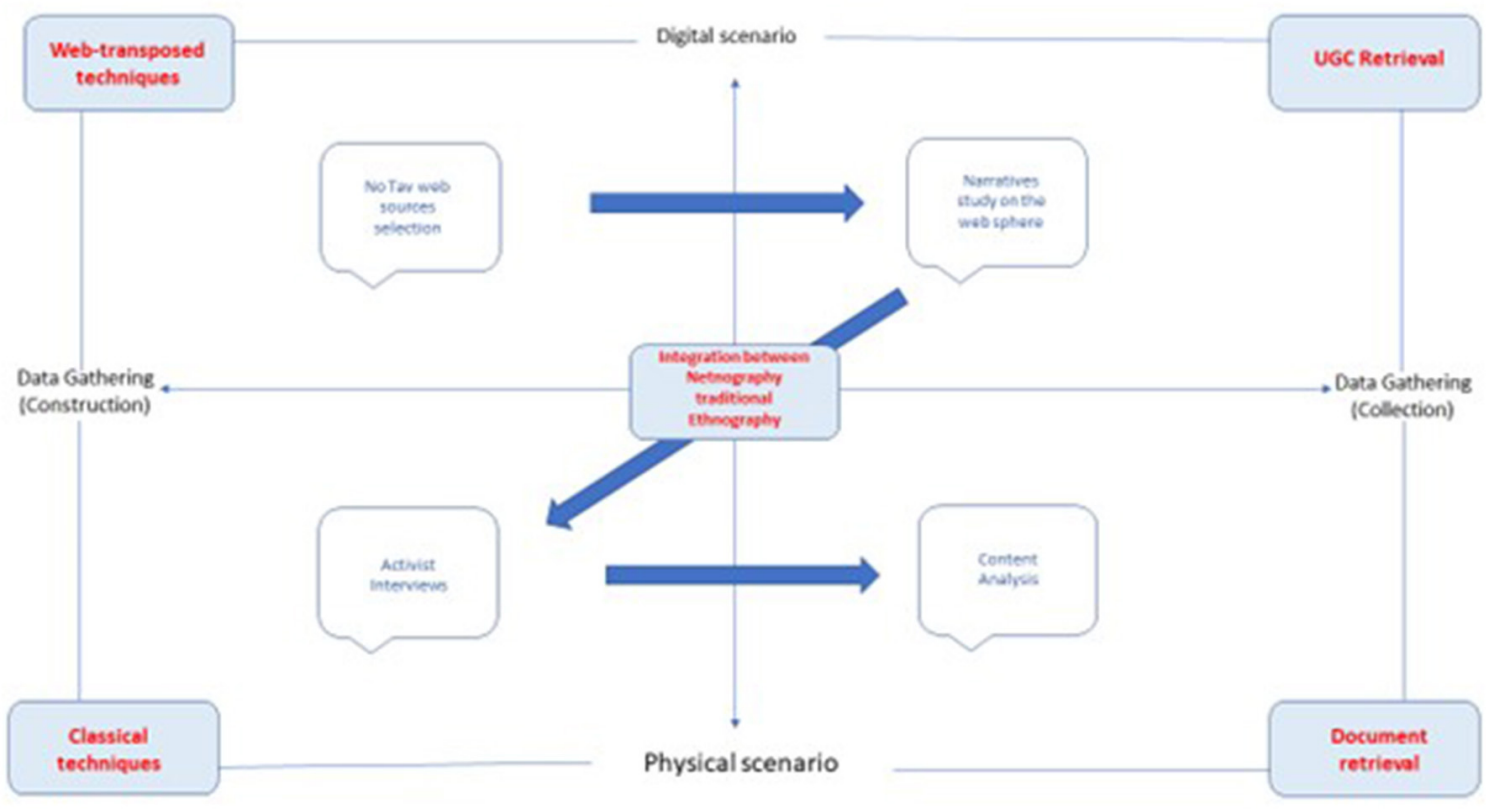
Typological scheme applied to *No Tav* study.

The integration among the multiple combinations of practices concerning either exogenous or endogenous features of technologies is turned into the best research plan to apply due to the main object of the study and its related research questions. It is the case of the abovementioned study by Padricelli (2023) on *the No Tav* Movement. In this case, as shown in Figure 3, the researcher who had the purpose of reconstructing the digital expression of a social movement by the longitudinal reconstruction of their relationship with the digital scenario was supported by the research question oriented to understand how the narration of activists changed over time due to the evolution of technical opportunities and, on the other hand, to comprehend the adoption of digital media in the daily life of activists. Considering this purpose, the researcher took the first position in the first quadrant, collecting all the cultural products posted in the main digital portions attended by *No Tav* Movement: Blogs and social media public pages. Although, the only result concerning the different use of blogs and social media for the movement claim was not enough to understand how activists adopted digital media because of the movement mobilization and organizational purposes. This is what emerged based on the results of a proper content analysis aimed at enlightening the main topics and narratives in the last 10 years.

For this reason, as shown in Figure 3, the researcher took the latter position in the third quadrant, meeting the activists on the physical field, and asking them about the opportunities and threats in the digital shift of their collective action in the last years. During this phase, the researcher asked at the same time for any documents valid to enrich the investigation, receiving some institutional documents, original communications, pamphlets, and pictures activists produced over the years. At the end of the integrated immersive-investigative experience, the obtained results gave the researcher exciting answers to the abovementioned research questions. These enlightened the progressive transformation of the role of digital media from a facilitating instrument to share information and diagnostic features concerning their claims, to an enabling way for mobilization aims and emancipating practices of communication (Milan, 2013) oriented to enlarge the movement network.

However, these connotations still leave one last point of interest in the shadows that it is important to recall. This point relates to the overall purpose of ethnographic study when elements connote different practices, methods, and ethnographic ways that the researcher applies in the field. Following the hybrid vocation of ethnographers who move inside and outside the digital scenario, the ways they access the field, take the position, and assemble data (Liu, 2022) allow researchers to practice a *reinforcement* of their immersive or investigative actions, combining different strategies of data gathering. This strategy can be pursued to emphasize the results of one method with those obtained by the other, clarify and shed light on particular results, or even expand visions and spans of action sequentially using different collection strategies. On the other hand, there is a different strategy, equally pursuable, which aims at a different goal, that of *integration of applications* supporting investigative or immersive practice inside and outside the digital environment, in order to overturn limits and fill the empirical disadvantages of the single application of the method.

## 4. Discussion and conclusion

### 4.1. The epistemic challenge

The increase of research based on ethnography over the years (as shown, among others, by Bartl et al., 2016; Heinonen and Medberg, 2018) connotes how the method has never diminished for social science and how ethnography continues to be the most popular way to observe and reflect on the daily social context. Regardless of the methodological context created by community, fieldwork, and role, the techniques' application shows how ethnography has duly transformed and adapted to the changes of the last few years. Relating to the case studies proposed, in which the authors have played a central role in doing research, the main discussion element underlined concerns a more complex move from the traditional application of ethnography to the netnographic method. On the other hand, the reference to the above case study aimed at enlightening the relevance of re-centered ethnography and mixing the various application opportunities depending on research questions, research purposes, and tools available.

In the wake of the reflections just conducted and trying to systematize a recognition of the vision of the complementary vocation of the method, it implies recalling an underlying perspective that moves transversally to them. It is the contribution of the focus of the innovation that moves from the side of the *innovation of the context* to the side of the *innovation in the method*, clearly showing that all scenario evolutions have implications for how knowledge is produced in that scenario. This is done by first testing the method in its classical application against a new cognitive challenge. When debates around this cognitive challenge and the evolution of the object, the web, are settled, it leads to the evolution of the method itself, creating innovation in the methods of investigation. This leads us to revisit and discuss the four main pillars subjected to epistemic challenges due to social change and methodological advances that are identifiable in

(1) the transformations of observable communities in terms of boundless scenarios (Kozinets, 2020; Nasciemnto et al., 2022);
(2) the changes that have taken place in terms of fieldwork in terms of multi-sited and short-term fieldwork (Seligmann and Estes, 2020);
(3) concerns innovations in the instruments and in the role of the researcher over time in terms of the hybridization of methods and disciplines (Seligmann and Estes, 2020);
(4) the scenario conceived in terms of agency as a restructuring of the concept of platform agency (Nasciemnto et al., 2022).

The first pillar implies for the researcher the impossibility of compartmentalizing the environments and mechanisms of influence, imposing more complex and profound interpretative logic, centered on and imbued with the digital. This assumption happens in the field elected as the context of ethnographic practice, the sometimes-uncontrollable fluidity that urges practices, objects, and subjects to be simultaneously inside and outside the digital scenario.

The second pillar follows the conception of changes that have taken place in terms of multisided and short-term fieldwork. Fieldwork can and should occur anywhere, even if that “site” stretches into multiple places. However, “doing good multisided fieldwork is challenging, especially if researchers seek to go beyond doing interviews to carry out a fine-grained participant observation. Researchers must follow unpredictable ‘chains' and ‘networks' and use their skills to persuade gatekeepers to provide access. It is hard enough to figure out the topography of power in one location, and multiple sites. This kind of research demands that researchers fully take advantage of contacts they have in order to persuade gatekeepers to permit them access” (Nasciemnto et al., 2022, p. 178–179). This particular perspective implies a total immersion in the values and meanings assigned to a subject in the different sites and requires time and broad, non-stereotypical, or researcher-centered knowledge. However, the demand for multi-situated interpretative and comprehension skills requires this onerous commitment of involvement, time, and interpretative skills. In that case, this is counterbalanced by the increasingly popular short-term fieldwork. This attitude in research practice has constantly developed on the digital scene, characterizing digital fieldwork in a very peculiar way. We have already mentioned that the classic field notes born from observation, field presence, and mediation of meaning through the researcher's reflections, are supported by the possibility of having access to a wide range of pre-existing, persistent, and coexisting data as the object of continuous interaction on the net, which are the digital traces. The wide availability of these materials helps to compress the long processes of ethnographic practice and challenges perhaps the most classic of the method's cornerstones.

The third pillar concerns innovations in the instruments and in the role of the researcher over time in terms of the hybridization of methods and disciplines (Seligmann and Estes, 2020). If the ethnographic method was characterized in its early days by being born in a precise disciplinary context of an anthropological nature, today, it turns out to be a method used by various disciplines, often referring to the need for interdisciplinary work in order to obtain the best possible result through this research practice. Disciplinary contaminations are reflected in the combination of different methods that give rise to innovative practices recognizable, for instance, in participatory field methods with collaborative ethnography, overshadowing the central role of the interpretive phase devoted to the ethnographer, or the use of interviews as ethnography, this time overshadowing the fact that interviews give a partial and reworked view of the subjects concerning the object of investigation, sacrificing that outsider's view of the ethnographer, now increasingly involved in digital scenarios. Nevertheless, what may appear to be limitations push toward what we have previously called reinforcing and integrating practices in the three turning points that Liu (2022) defined as access to the field, taking the position, and assembling data. However, if observation, interrogation, and reading, are classically conceived as the main actions with which the researchers can produce data on social phenomena, nowadays it is possible to observe a progressive transition from traditional techniques unrelated to the context in which they are applied to a unique hybrid with the same entire context overcoming the concept of space where cultures can be studied (Woolgar, 1996). Ethnographers moved to an all-encompassing environment, rediscussing the notion of community and fieldwork norms in a reshaping of the researchers' role. The digital scenario, therefore, shows how ethnography is today shaped in several directions drawn by footprints left behind on the various paths walked by users to express identities and values on the Internet and to build their relational networks to share knowledge. The current framework cannot be intended as a final frame, nor projected onto further changes or drastically detached by limitations or criticism. Today, the sociological debate must review its epistemological profile to comprehend not only how ethnographic methods can coexist or be isolated for specific applications in research but also prompt discussion of its ontological basis. On the one hand, it is possible to assume how the digital scenario and the evolution of no-intrusive observation techniques oriented to gather discourse and spontaneous traces left by users allow researchers to overcome the critical opposition between modernists and postmodernists to reflect on the neutral vocation of ethnographers. On the other hand, it became more and more evident that it is necessary to consider if and how the hybridization of techniques and social context is intended, still speaking, in methodological terms, of pure ethnography, or maybe assuming the digital scenario as a whole environment open to the entirety of research methods in social science involving ethnography and computational and data science.

The last pillar focuses on the scenario not as a passive context, a scene on which action, interaction, and reaction of objects and subjects take place, but rather, the scenario is conceived in terms of scenario agency as a restructuring of the concept of platform agency (Nasciemnto et al., 2022). It opens another interesting opportunity for reflection and empirical opportunities to understand the assumptions related to the ethnographic practice that classically recognizes the agency of the individual by analyzing how the contexts and scenarios within which they move to shape and impact their identities, practices, and interactions. In this way, further studies must be addressed to comprehend how the digital scenario, the use of digital devices, and its characteristics can shape the possibilities of individual agency by creating an infrastructure that acts on the subjects and the possibilities of the individual's agency. Therefore, on this pillar: *how does the scenario change the mechanism of influence, determination, and co-construction?*

The idea that the current methodological shape in ethnography is open to other critical limitations is already highlighted by scholars (Addeo et al., 2021; Murphy et al., 2021). Reflections on research methods can be sure of the unquestionable assumption: *ethnography never stops but undoubtedly, today it can no longer be considered the same as in the pre-digital era*. Despite continuing interest in the method, there are undeniable evolutions. Rather than referring to fashions of the moment, they indicate a progressive growth of both the object of study and the method used to study it.

At first, the ethnographic method was used to approach the study of a series of phenomena that also assumed a digital form that neither remains unchanged nor slowly changes. However, instead, it undergoes different accelerations as society progresses. Therefore, the ethnographic method, which configures slow, prolonged, and in-depth research paths, must begin to follow the object more closely and modify itself in the function of a better adaptation to it. Moreover, here is where the reflection on the method in the classical disciplines returns to the stage, perceived as transposed to a new place. In this new place, evolutions continue to progress over time, from Web 1.0 to a concept of the Web that is increasingly relational, with Web 2.0, and interacts with all the subsequent evolutions. On the Internet, there is not only a new context or place to which the ethnographic method can be adapted, but also the transposition of society and social structures that encompass identities, values and, with time, also defined interests, leading the ethnographic method in the digital context to involve other disciplines and increasingly specific arguments.

Therefore, the evolution does not stop, and the innovative path moves among these joint changes that work toward the level of theory and approaches, the production of knowledge, and constant questioning of the future of ethnographic research. Especially now that the ethnographer is not only socialized but is also a real native of hybridized digital scenarios in which persistent traces are found, collectible and reworkable beyond the issues of opening up the field, sharing and seeking feedback, reflexivity, and subjectification of the research. This made the method recognizable in its early days and ensured that it retained its importance and relevance despite the changes occurring in the digital age.

Perhaps an obvious but necessary conclusion remains an attempt to show how, despite all the changes, hybridizations, and contaminations, the ethnographic method resists firmly in its univocal soul under whose umbrella it manages to shape the different forms in which its applications and new directions present themselves.

## Data availability statement

The original contributions presented in the study are included in the article/supplementary material, further inquiries can be directed to the corresponding author.

## Author contributions

Although the manuscript is the result of joint work, section 2 and 3 are to be attributed to GMP, and section 1 and 4 to GP. All authors contributed to the article and approved the submitted version.
